# Calculations of BODIPY dyes in the ground and excited states using the M06-2X and PBE0 functionals

**DOI:** 10.1007/s00894-016-3108-8

**Published:** 2016-10-07

**Authors:** Marina Laine, Nuno A. Barbosa, Robert Wieczorek, Mikhail Ya. Melnikov, Aleksander Filarowski

**Affiliations:** 1 Faculty of Chemistry, University of Wroclaw, F. Joliot-Curie 14, Wroclaw, 50-383 Poland; 2 Department of Chemistry, Moscow State University, F. Joliot-Curie 14, Moscow, Russia; 3 Department of Physics, Industrial University of Tyumen, 625-000 Tyumen, Russia

**Keywords:** DFT, TD-DFT, BODIPY

## Abstract

A number of fluorescent dyes based on BODIPY (4,4′-difluoro-4-bora-3a,4a-diaza-*s*-indacene) have been studied theoretically. This paper presents the results of calculations of these BODIPY dyes in their ground and excited states, performed using DFT and TD-DFT methods, respectively. The influences of *N*,*N*-dimethylaminobenzyl, *ortho*-fluorophenol, and methyl substituents as well as the solvent polarity on the positions of the absorption and emission bands of the dyes were analyzed. The computational data obtained in this work were compared to the corresponding experimental data. The trends in the experimental data were found to agree with those shown by the computational data. Differences between the potential curves obtained when using linear-response (LR) and state-specific (SS) approaches for the ground and excited states are also reported.

**Electronic supplementary material:**

The online version of this article (doi:10.1007/s00894-016-3108-8) contains supplementary material, which is available to authorized users.

**Graphical Abstract:**
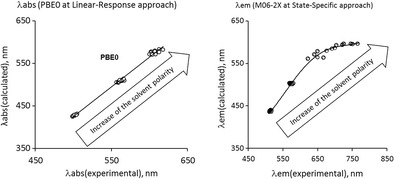
The article shows that the trends of the experimental dependencies λ_abs_ = f(Δf) and λ_em_ = f(Δf) well described by PBE0 (LR approach) and M06-2X (SS approach) calculations, respectively. The influence of substituents on the spectral characteristics of the BODIPY chromophore are analysed

## Introduction

This paper presents a quantum-mechanical study of fluorescent dyes based on 4,4′-difluoro-4-bora-3a,4a-diaza-*s*-indacene (BODIPY) [1]. Photophysical studies of these compounds and the design of novel compounds of this type have become very popular over the last decade [2–4]. Such interest is due to the ability to efficiently model the spectral properties of BODIPY derivatives obtained by introducing different types of substituents onto the 4,4′-difluoro-4-bora-3a,4a-diaza-*s*-indacene core, or by modifying the core in other ways [5]. These compounds are also popular due to their potential application in optoelectronics [6], medicine [7], and biology [8]. Indeed, laborious quantum-mechanical calculations of BODIPY dyes in the excited state using time-dependent density functional theory (TD-DFT) methods are being applied more and more [9–17]. The use of the PBE0 and M06-2X functionals in such studies is motivated by the investigations presented in [14–17].

The task of the work reported in this paper was to computationally study the effects of solvents of various polarities, and the impact of introducing different substituents onto the BODIPY core, on the positions of the absorption (S_0_→S_1_) and emission (S_1_→S_0_) bands of dyes based on BODIPY using TD-DFT with the PCM approach. The substituents chosen for this study were selected because we obtained relevant experimental data on the corresponding BODIPY derivatives in our previous work [18]. We were able to theoretical derive an accurate description of these dyes, which will make it possible to model such dyes and to determine the nature of the dye–environment interrelations, as well as to identify novel dyes with required spectral characteristics.

## Computational details

The calculations were carried out with the Gaussian09 program [19]. The PBE0 [20] and M06-2X [21] functionals and the 6-31 + G(d,p) basis set [22–31] were used for calculations. The structures of the studied compounds were optimized in the ground (S_0_) and excited (S_1_) states by DFT [32, 33] and TD-DFT [34, 35] methods, respectively. The absorption (i.e., the S_0_ → S_1_ transition) and emission (the S_1_ → S_0_ transition) bands were calculated for the compounds in the gas phase and in various solvents with a range of polarities. The absorption spectra were obtained by calculating the first six low-lying excited states within the vertical linear-response, nonequilibrium TD-DFT approximation. The state-specific nonequilibrium solvation approach was applied by saving the solvent reaction field from the ground state. Solvent effects in the ground and excited states were taken into account with the polarizable continuum model [36–38] (PCM), using the integral equation formalism variant (IEFPCM) as a default SCRF method. The states with the largest oscillator force values were optimized in the excited state, and emission spectra were calculated within a linear-response, equilibrium TD-DFT approximation and using state-specific non-equilibrium solvation. The contribution of solvent effects was computed with both the linear-response (LR) [39, 40] and state-specific (SS) [41] quantum mechanical approaches. The selected data and structures are collected in Tables S1–S4 of the “Electronic supplementary material” (ESM).

## Results and discussion

### Impact of the substituents

**Scheme 1:**
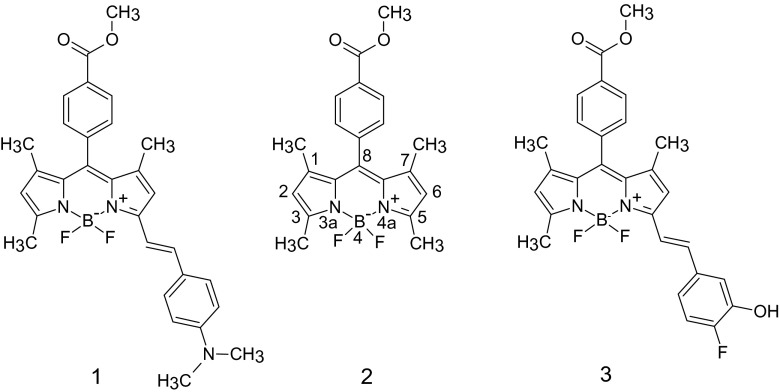
Structures of the studied compounds

As stated in the “Introduction,” the aims of this work were to verify the data afforded by TD-DFT and DFT methods for the dyes of interest in a wide range of solvents (ranging from hexane to DMSO), and to analyze the impact of substitution at position 5 of the BODIPY core with various substituents on the positions of the absorption and emission bands of the dyes. To achieve these aims, three compounds based on BODIPY were studied (Scheme 1).

**Fig. 1:**
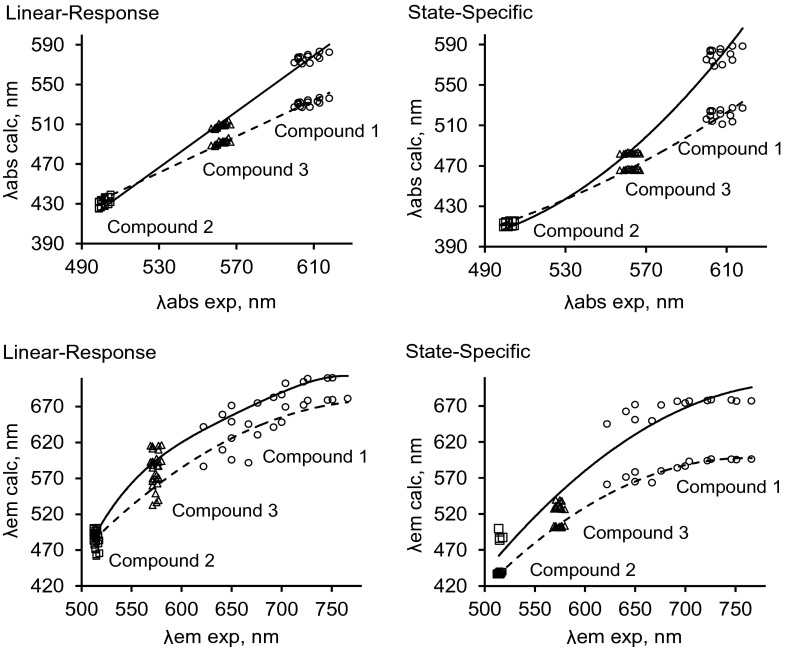
Comparison of the data for the parameters *λ*_abs_ and *λ*_em_ calculated for the compounds of interest using the PBE0 (*solid line*) and M06-2X (*dashed line*) functionals in linear-response (LR) and state-specific (SS) approaches with the corresponding data for *λ*_abs_ and *λ*_em_ obtained experimentally

These compounds were selected because a considerable amount of experimental data on them is available [18]. Compound **2** plays the role of a reference compound in which a methyl group has been inserted at position 5 (*σ*_R+_ = −0.08 [42]). For compounds **1** and **3**, *N*,*N*-dimethylaminobenzyl and *ortho*-fluorophenol moieties, respectively, were added at position 5 instead of the methyl group. According to computational and experimental data, the *N*,*N*-dimethylaminobenzyl substituent has the strongest effect on the positions of the absorption and emission bands (i.e., the largest bathochromic shift), which is due to the strong electronic effect [42] of this substituent. However, although the *ortho*-fluorophenol moiety of **3** has a weaker electronic impact than the *N*,*N*-dimethylaminobenzyl fragment, **3** does still show bathochromic shifts in the absorption and emission bands with respect to **2**. Note that the data calculated using the PBE0 and M06-2X functionals show similar trends to the experimental data (see Fig. 1 and Fig. S1 in the ESM).

Based on the results of the calculations and the corresponding experimental data, we can conclude that the total electronic effect of the –CH=CH–(fluorophenol) substituent is much stronger than the electronic effect of the methyl group (*σ*_R+_(CH=CH_2_) = −0.16, *σ*_R+_(C_6_H_5_) = −0.22, *σ*_R+_(F) = 0.34, and *σ*_R+_(OH) = 0.12; all data from [42]); the same is true when the –CH=CH–(*N*,*N*-dimethylaminobenzyl) substituent is compared to the methyl substituent (*σ*_R+_(CH=CH_2_) = −0.16, *σ*_R+_(C_6_H_5_) = −0.22, and *σ*_R+_(N(CH_3_)_2_) = −0.64; all data from [42]). When the –CH=CH–(fluorophenol) substituent is present, the hydroxyl group of the substituent has only a minor π-electronic effect on the BODIPY core because the hydroxy group is *meta* with respect to the –CH=CH– bridge. The π-electronic effect of the fluorine atom present on this substituent is also not significant. Indeed, the M+ and M− effects exerted by the fluorine atom and the hydroxyl group cancel each other out to some extent, weakening their influence on the BODIPY core. Therefore, the moiety that has the greatest influence on the bathochromic shifts of the absorption and emission bands of compound **3** with respect to compound **2** is the –CH=CH–C_6_H_5_ fragment (*σ*_R+_(CH_3_) = −0.08, *σ*_R+_(–CH=CH_2_) = −0.16, *σ*_R+_(−C_6_H_5_) = −0.22; all data from [42]).

The substituent that has the strongest effect on the spectral parameters is found to be –CH=CH–(*N*,*N*-dimethylaminobenzyl). This substituent causes significant bathochromic shifts of the absorption and emission bands (Δ*λ* ∼ 100–200 nm) for compound **1** with respect to those of compounds **2** and **3**. This phenomenon is due to the strong π-electronic effect of the *N*,*N*-dialkylamine group on the spectral characteristics of compound **1**, which is described in detail in [43, 44].

All of the calculations performed in this work utilized the two most reliable functionals for boron compounds: PBE0 and M06-2X [45, 46]. A comparison of the data provided by the PBE0 and M06-2X functionals when using the LR approach with the corresponding experimental data shows that the calculated absorption bands are the closest to the experimentally determined absorption bands for compound **1**. However, compound **1** also shows the largest deviation between the results obtained using the PBE0 and M06-2X functionals; the deviation for compound **3** is smaller, and there is almost no deviation for compound **2**. Both PBE0 and M06-2X give almost the same positions for the absorption bands of compound **2** (Fig. 1). It is also clear that the results obtained using the SS approach (Fig. 1) are more accurate (i.e., they deviate less from the corresponding experimental data) than those yielded by the LR approach. However, there are some problems with the trend in absorption band shifts obtained using the SS approach. The computational results obtained with the LR approach exhibit a satisfactory trend regardless of the functional used (Fig. 1), whereas the results obtained using the SS approach suggest that increasing the solvent polarity causes very small changes in *λ*_abs_ for compounds **2** and **3** , which is not correct. Increasing the solvent polarity actually increases the bathochromic shifts of the absorption bands, but this phenomenon is barely apparent in the data obtained using the SS approach with both functionals (PBE0 and M06-2X) (Fig. 1).

**Fig. 2:**
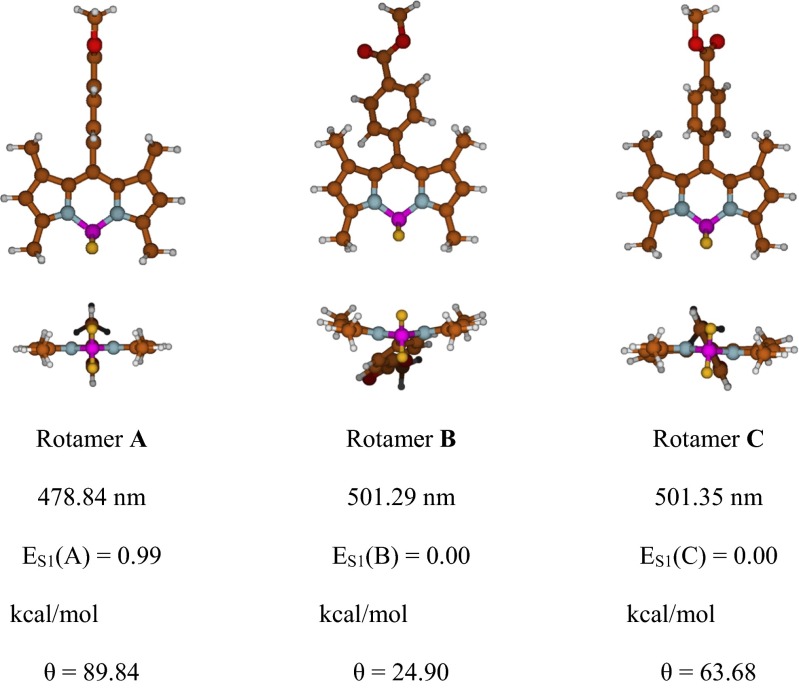
Structure of compound **2** in the excited state in DMSO, as calculated using the PBE0 functional

Before comparing the calculated emission band data with the corresponding experimental data, a conformational analysis was performed. Three rotamers with similar potential energies were calculated for compound **2** in the excited state (Fig. 2). These rotamers differ in the position of the phenyl ring at the *meso* position. In the first rotamer (**A**), which is also the least stable, the phenyl ring is almost perpendicular to the core of the BODIPY chromophore, whereas the phenyl ring is significantly twisted in the second (**B**) and third (**C**) rotamers (*θ*_A_ = 89.8°; *θ*_B_ = 24.9°; *θ*_C_ = 63.6°). It should be pointed out that the position of this phenyl ring is determined by the relative strengths of two contradictory effects: (1) a steric effect between the phenyl ring and the two methyl groups at positions 1 and 7, which causes the phenyl ring to attempt to adopt a perpendicular position, and (2) π-electronic coupling between the core of the chromophore and the phenyl ring, which leads to a flattening of the molecule.The steric effect predominates in the excited state, resulting in a decrease in the energy of the molecule of 0.99 kcal/mol. Choosing a less stable conformer can lead to an error of about 22 nm in the position of the emission band. The second (**B**) and third (**C**) rotamers possess almost the same energies, and the positions of their emission bands are also very similar. However, the structures of these rotamers are different: the torsion angle of the phenyl ring is 24.9° for rotamer **B** and 63.6° for **C**. Therefore, the core of the chromophore is flat in **C** and curved in **B**. The abovementioned results suggest that it is possible to twist the phenyl ring to transition from one rotamer to another, and that bending the chromophore core leads to a transition from rotamers **B** and **C**: Δ*E*_A-B_ = *E*_S1_(**A**) – *E*_S1_(**B**); Δ*E*_A-C_ = *E*_S1_(**A**) – *E*_S1_(**C**) = 0.99 kcal/mol. Based on the results of the conformational analysis, conformer **C** was used in subsequent calculations.

It is important to underline that the calculations performed using the LR approach overestimated the shifts in the emission bands for compounds **2** and **3** (Fig. 1) that occur upon increasing the solvent polarity. In terms of the slope of the curve, the data for compound **1** are the closest to the corresponding experimental emission band data. As also seen for the absorption bands, the positions of the emission bands obtained by the PBE0 method are closer to the corresponding experimental data (630–700 nm for the calculated data vs 640–720 nm for the experimental data; Fig. 1) than the data obtained by the M06-2X method (590–670 nm for the calculated data vs 640–720 nm for the experimental data) when using the LR and SS approaches. However, the correlation between the data and the trend line is better when using M06-2X than when using PBE0. The calculations performed for compounds **2** and **3** using both the M06-2X and PBE0 functionals with the LR approach overestimate the influence of the solvent: note that the data points for each compound in the bottom left plot of Fig. 1 (which represent data obtained for the same compound in various solvents) are distributed in an almost vertical line, indicating that varying the solvent has a far greater effect on the calculated value of *λ*_em_ for a particular compound than on the corresponding experimental value. On the other hand, when the SS approach is used with either functional, increasing the polarity of the solvent leads to relatively small shifts in the emission bands for compounds **2** and **3**, which agrees well with the trend seen in the corresponding experimental data. The shifts seen in the calculated data for compound **1** are only in satisfactory agreement with the corresponding experimentally observed shifts, however.

### Analysis of the influence of the solvent on the spectral characteristics of the dyes

**Fig. 3:**
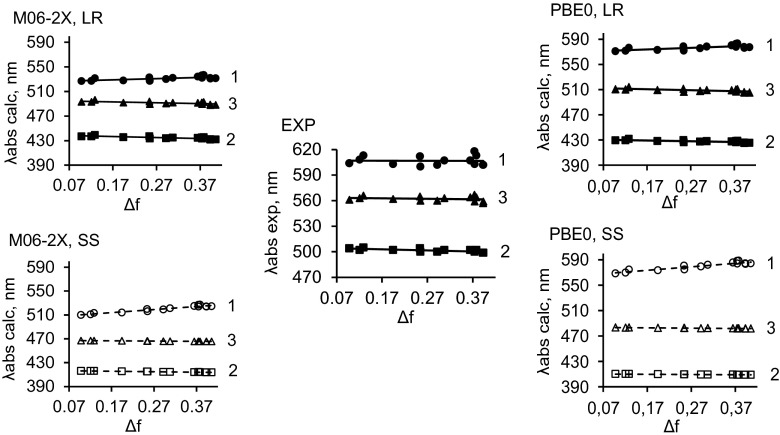
Experimental (*graph in the center*) and calculated *λ*_abs_ values of compounds **1**–**3** versus the Lippert solvent parameter ∆*f* (see Eq. 1)

**Fig. 4:**
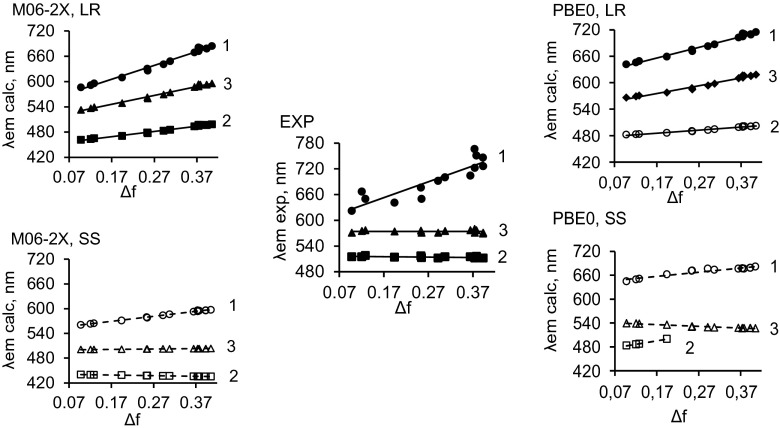
Experimental (*graph in the center*) and calculated *λ*_em_ values of compounds **1**–**3** versus the Lippert solvent parameter ∆*f* (see Eq. 1)

To describe the influence of the solvent on the positions of both the absorption and emission bands calculated by the TD-DFT method, the dependencies of *λ*_abs_ and *λ*_em_ on the Lippert solvent parameter Δ*f* were examined (see Figs. 3 and 4). The Lippert–Mataga parameter can be defined as [48–50]

1 $$ \varDelta f = f\left(\varepsilon \right)\hbox{--} f\left({n}^2\right)=\left(\varepsilon \hbox{--} 1\right)/\left(2\varepsilon +1\right)-\left({n}^2\hbox{--} 1\right)/\left(2{n}^2+1\right), $$

where the parameters *ε* and *n* are the dielectric constant and the refractive index of the solvent, respectively.

These relationships (derived using the SS and LR approaches) between the positions of the absorption and emission bands and Δ*f* reflect the interactions between ground- and excited-state dye and solvent molecules. According to previous reported experimental data [18], significantly increasing the solvent polarity (i.e., changing the solvent from hexane to DMSO) does not cause a visible shift in the absorption bands of the compounds of interest (see the graph labeled EXP in Fig. 3).

Remarkably, the TD-DFT calculations also indicate that shifts in the absorption bands with increasing solvent polarity are rather small (regardless of whether the PBE0 or M06-2X functional and the SS or LR approach are used; Fig. 3). This effect indicates that significant structural changes (e.g., proton transfer, isomerization, etc.) are absent in the ground state. On the other hand, replacing the substituent (i.e., **2** → **3** → **1**) strongly influences the position of the absorption band, causing a bathochromic shift of this band (Fig. 3). As mentioned above, this phenomenon is caused by differences in the electron-donor strengths of the substituents. The order of absorption-band bathochromic shifts (compound **1** > **3** > **2**) obtained using TD-DFT calculations is in good agreement with the experimentally observed order [18]; see the graphs in Fig. 3. However, the TD-DFT calculations performed using the SS approach with both functionals [see the (M06-2X, SS) and (PBE0, SS) graphs in Fig. 3] overestimate the influence of the solvent polarity on the position of the absorption band of compound **1**.

When it is used to calculate the influence of the solvent on the position of the emission band, the LS approach (with both functionals) indicates that there is a systematic bathochromic shift in the emission band of compound **1** with increasing solvent polarity (i.e., increasing Δ*f*; Fig. 4), but not for compounds **2** and **3**. The dependency of *λ*_em_ on Δ*f* was illustrated more precisely by applying the M06-2X functional and the SS approach. Increasing solvent polarity led to small changes in the positions of the emission bands for compounds **2** and **3** and a significant bathochromic shift for compound **1**. Thus, to sum up, calculations performed using the LR approach (with both functionals) gave more accurate results (i.e. data closer to the corresponding experimental data) than the SS approach, although the data obtained using the SS approach (with the M06-2X functional) showed more precise trends. Notably, the LR and SS approaches yield different potential curves in the ground and excited states. The calculations performed using the LR approach led to wider potential curves for the ground and excited states than those obtained using the SS approach (Fig. S2 in the ESM).

It is important to note that the solvent polarity parameter *E* ^N^_T_(30) more accurately describes hydrogen bonds than the parameter Δ*f* does; see [47]. When we explored the dependency of the Stokes shift Δ*ν* on *E* ^N^_T_(30), we obtained rather complicated results, because it was necessary to account for the polarity of the solvent before the positions of the absorption and emission bands could be determined accurately.

**Fig. 5:**
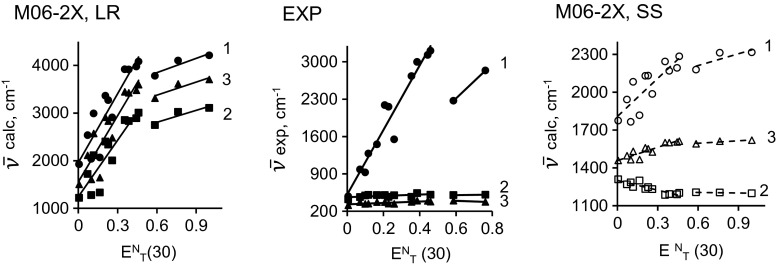
Dependency of the Stokes shift on the solvent polarity parameter *E* ^N^_T_(30) for each of the studied compounds

The experimental dependency reported in [18] shows that for compounds **2** and **3**, the Stokes shift remains almost constant with increasing solvent polarity (see the EXP graph in Fig. 4). This phenomenon indicates that the molecule is weakly polarized during the transition from the ground state to the excited one. However, the Stokes shift is observed to increase significantly for compound **1** with increasing solvent polarity. That said, these changes in the Stokes shift are not stable, because the Stokes shift value for compound **1** decreases notably when this compound is placed in a protic solvent. This decrease in Stokes shift is the result of hydrogen bonding between the *N*,*N*-diethylamine moiety and the protic solvent. These interactions reduce the effect of the π-conjugated coupling between the *N*,*N*-diethylamine fragment and the BODIPY core, which, in turn, leads to an increase in the polarization of the dye and thus an increase in the Stokes shift. Unfortunately, not all of the calculations give results that agree well with the corresponding experimental results. The calculations performed using the M06-2X functional with the LR approach reveal sudden decreases in the Stokes shift for all three compounds (Fig. 5). However, satisfactory results were obtained using the M06-2X functional with the SS approach.

We also report here on the difference between the potential curves obtained using the SS and LR approaches for the ground and excited states (Fig. S2 in the ESM). We found that that potential curves obtained using the SS approach were wider for the ground and excited states than those obtained using the LR approach.

## Conclusions

This paper presents a comparison of the results of calculations of BODIPY dyes performed using the M06-2X and PBE0 functionals and the LR and SS approaches with the corresponding experimental data.

An analysis of the influence of the substituent (either *N*,*N*-dimethylaminobenzyl, *ortho*-fluorophenol, or methyl) at position 3 of the BODIPY core was accomplished. It was found that the presence of the *N*,*N*-dimethylaminobenzyl substituent induced the largest shifts of the absorption and emission bands of the dyes to the bathochromic region, in good agreement with the experimental data [18, 51, 52].

A conformational analysis of the studied compounds in the excited state was performed, based on TD-DFT calculations. It was found that the three identified conformers do not differ significantly in energy. A similar conclusion was drawn by Mennucci and coworkers [53] following calculations of BODIPY dyes performed using the LR and cLR approaches.

The positions of the absorption and emission bands calculated using the PBE0 and M06-2X functionals with both approaches tend to show bathochromic shifts, where the shifts increase according to the sequence: compound **2** < compound **3** < compound **1**, which is in accordance with the experimental results. As for the dependencies of the spectral parameters (*λ*_abs_ and *λ*_em_) on the Lippert solvent parameter Δ*f*, we found that (1) the trend for *λ*_abs_ vs Δ*f* observed experimentally is accurately reproduced by calculations utilizing the PBE0 and M06-2X functionals and the LR approach (the SS approach provides only satisfactory results), and (2) the trend in *λ*_em_ vs Δ*f* observed experimentally is well described by calculations performed using the M06-2X functional and the SS approach. It is a remarkable fact that only calculations carried out using the M06-2X functional and the SS approach were able to describe the dependency of Δ*ν* on the solvent polarity parameter *E* ^N^_T_(30) even fairly well. Therefore, the influence of the solvent on the absorption and emission bands of the dyes of interest should be determined using two functionals: the PBE0 functional (with the LR approach) and the M06-2X functional (with the SS approach).

## Electronic supplementary material

Below is the link to the electronic supplementary material.

ESM 1 (DOCX 2261 kb)
