# Supplementary material for: Calculations of BODIPY dyes in the ground and excited states using the M06-2X and PBE0 functionals
Source: J Mol Model. 2016 Oct 7;22(11):260. doi: 10.1007/s00894-016-3108-8 (PMC5054050; doi:10.1007/s00894-016-3108-8)
Supplement: Supplementary file 1 — (DOCX 2261 kb) [file 894_2016_3108_MOESM1_ESM.docx]

Calculations of BODIPY dyes in the ground and excited states by M06-2X and PBE0 functionals

Marina Laine,^1^ Nuno A. Barbosa,^1^ Robert Wieczorek,^1^ Mikhail Ya. Melnikov^2^ and Aleksander Filarowski^*1, 3^

Correspondence to: Aleksander Fialrowski *E-mail:* [*aleksander.filarowski@chem.uni.wroc.pl*](mailto:aleksander.filarowski@chem.uni.wroc.pl)

*Faculty of Chemistry, University of Wroclaw, F. Joliot-Curie 14, Wroclaw 50-383, Poland*

*Department of Physics, Industrial University of Tyumen, 625-000, Tyumen, Russia*

*E-mail:* [*aleksander.filarowski@chem.uni.wroc.pl*](mailto:aleksander.filarowski@chem.uni.wroc.pl)

| Table S1. Calculated structures, spectral and energetic parameters of studied compounds by PBE0 and M062X methods in gas. | | | | | | |
| --- | --- | --- | --- | --- | --- | --- |
|  | PBE0 | | | M062X | | |
|  | Ground state | Excited state | | Ground state | Excited state | |
| E, Hartree | -1698.87114 | -1698.86477 |  | -1700.10055 | -1700.09587 |  |
| λmax , nm | 528.52 | 611.04 |  | 491.81 | 545.32 |  |
| 1 compound | 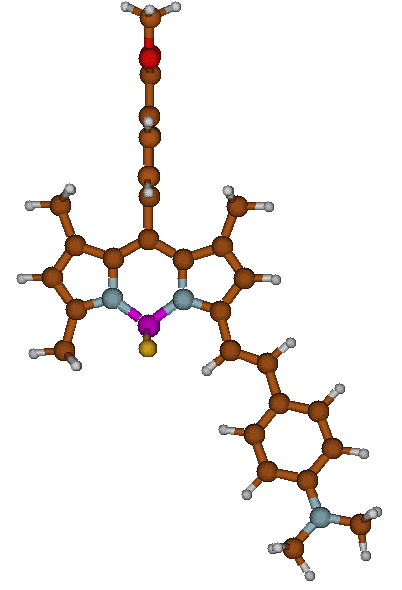 | 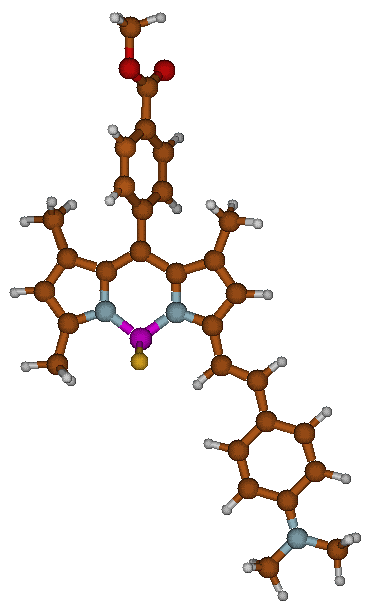 |  | 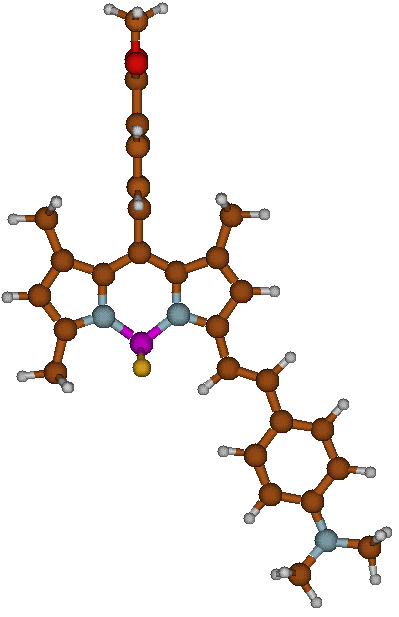 | 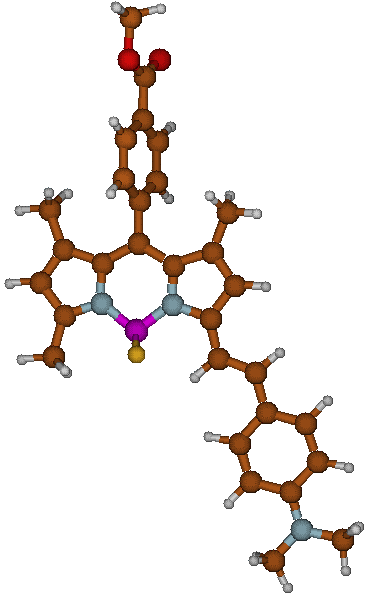 |  |
|  | 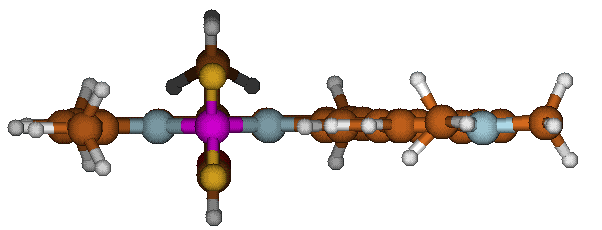 | 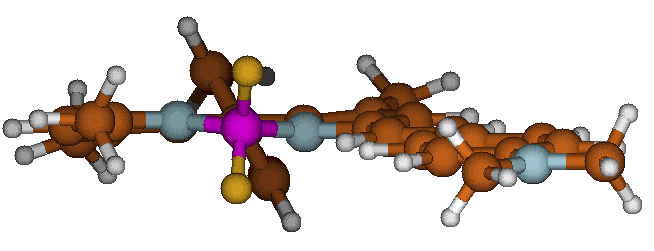 |  | 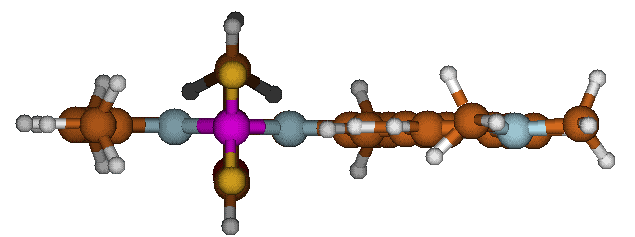 | 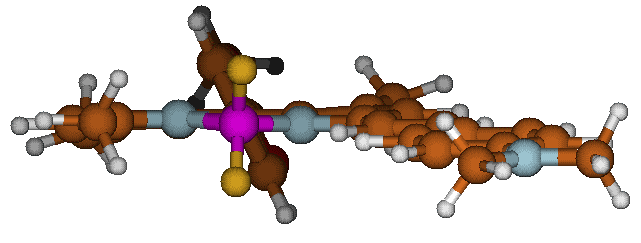 |  |
| E, Hartree | -1296.20891 | -1296.20099 |  | -1297.14081 | -1297.13709 |  |
| λmax, nm | 410.35 | 475.21 |  | 416.85 | 445.36 |  |
| 2 compound | 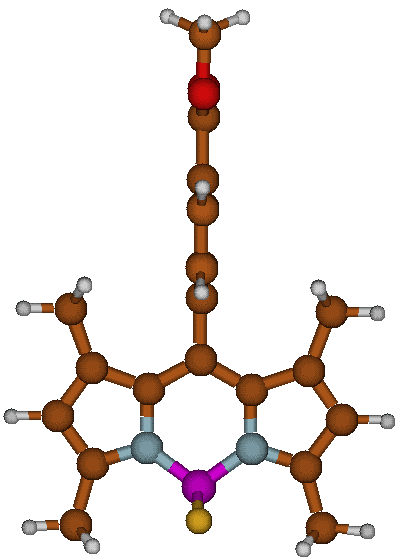 | 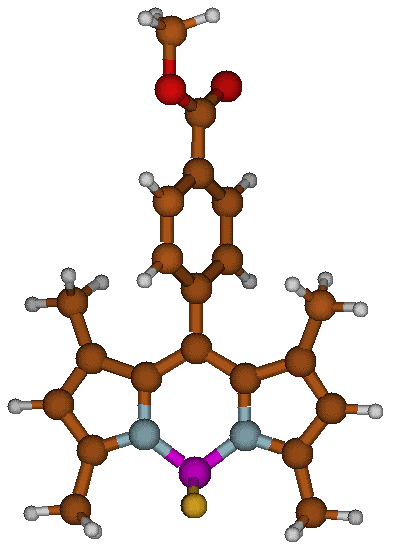 |  | 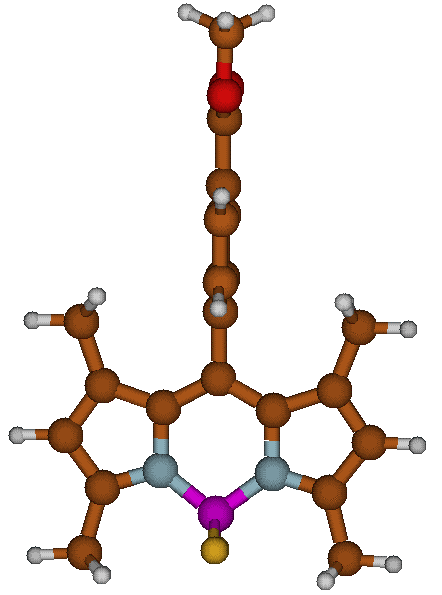 | 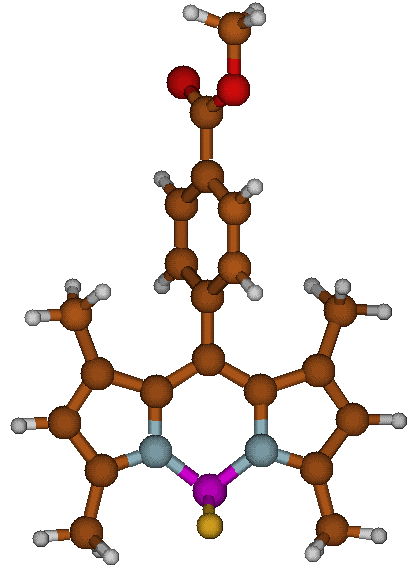 |  |
|  | 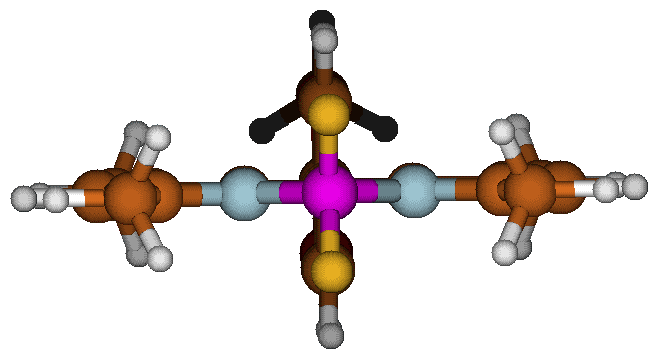 | 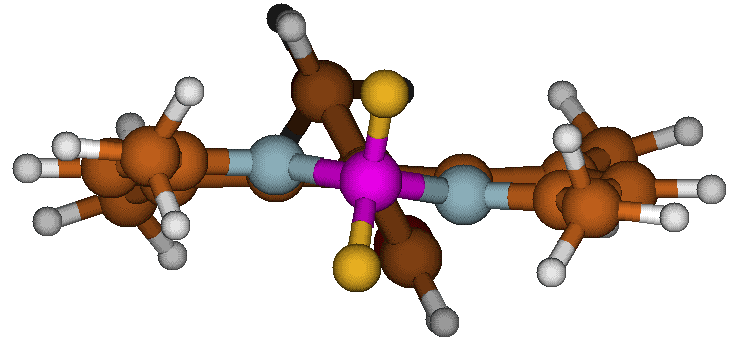 |  | 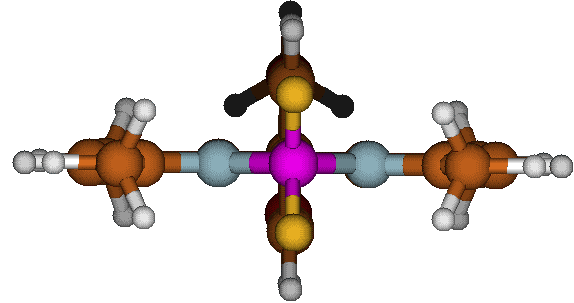 | 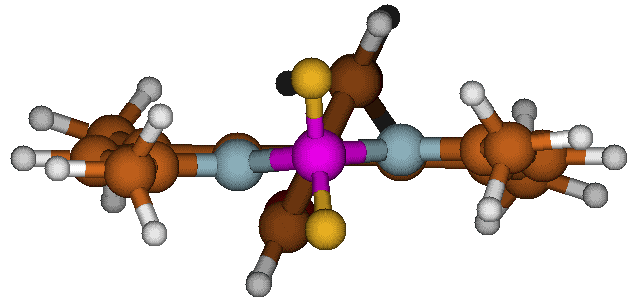 |  |
| E, Hartree | -1739.35332 | -1739.34704 | -1739.34693 | -1740.59744 | -1740.59357 | -1740.59372 |
| λmax, nm | 481.81 | 544.97 | 545.38 | 466.71 | 503.14 | 502.54 |
| 3 compound | 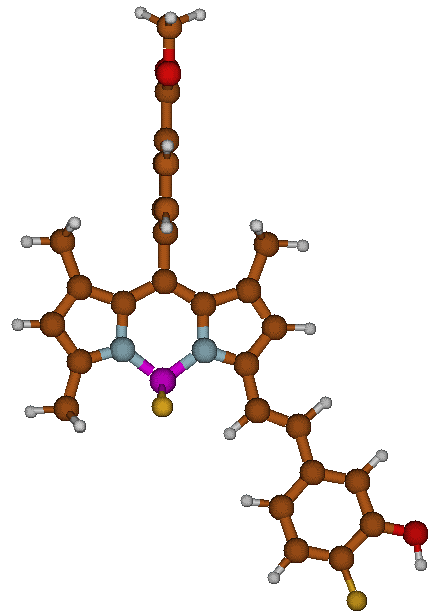 | 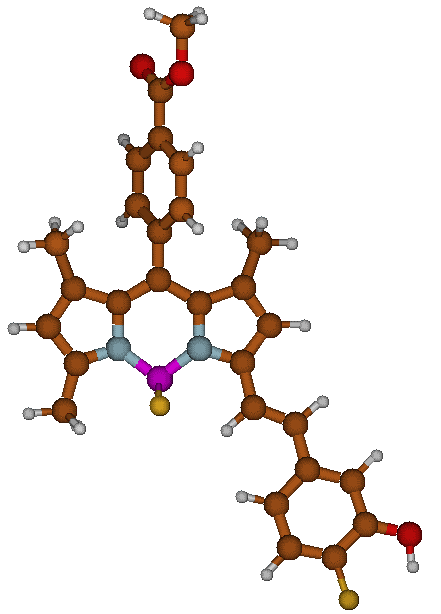 | 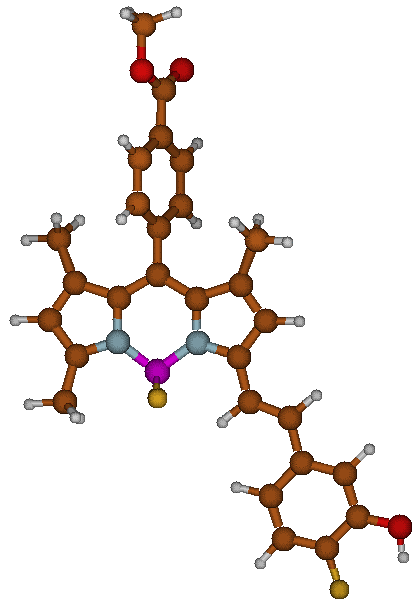 | 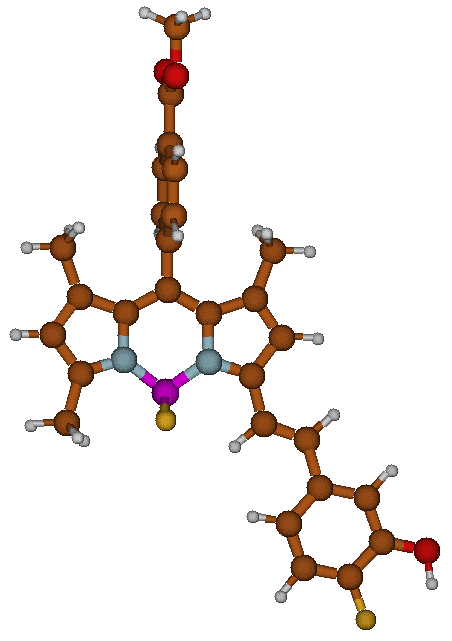 | 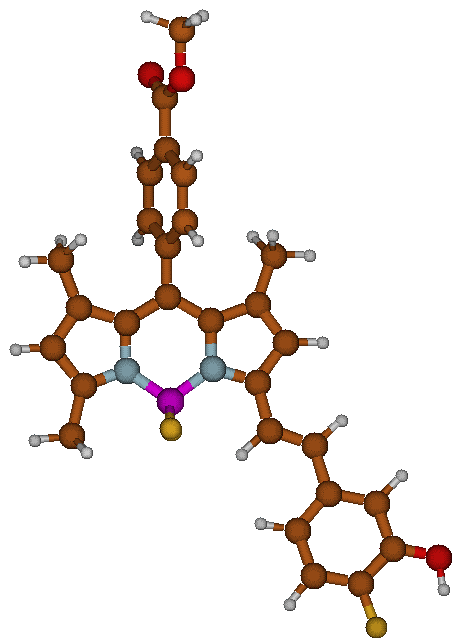 | 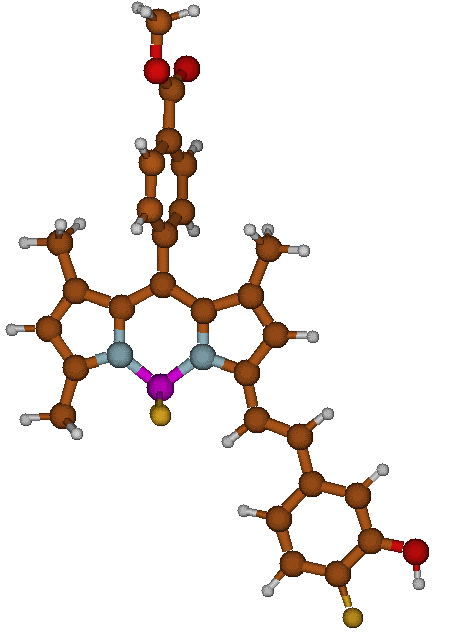 |
|  | 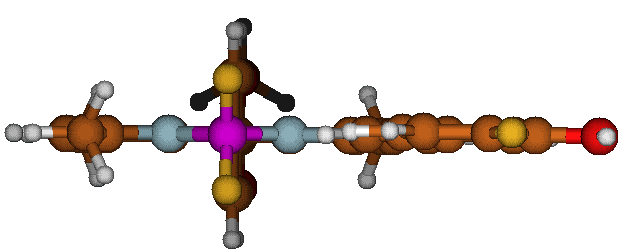 | 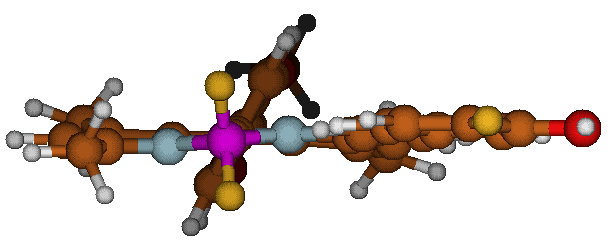 | 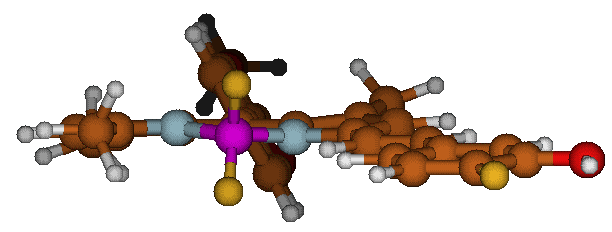 | 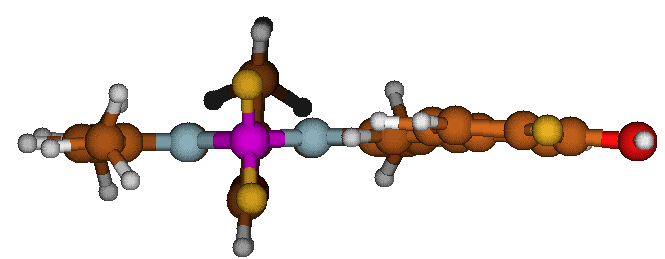 | 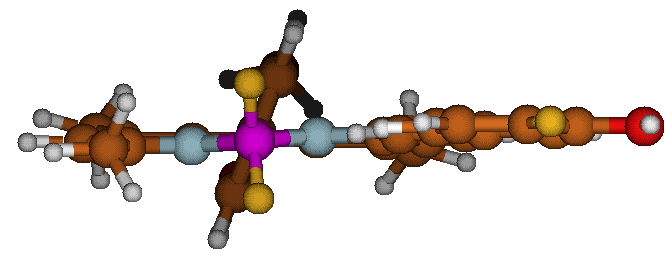 | 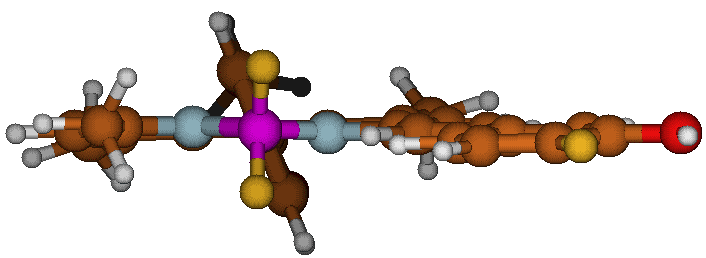 |

| Table S2. Calculated structures, spectral and energetic parameters of studied compounds by PBE0 and M062X methods in DMSO. | | | | | | | | |
| --- | --- | --- | --- | --- | --- | --- | --- | --- |
|  | PBE0 | | | | M062X | | | |
|  | Ground state | Excited state | | | Ground state | Excited state | | |
| E, Hartree | -1698.89313 | -1698.88945 |  |  | -1700.12189 | -1700.11662 |  |  |
| λmax, nm | 582.35 | 711.22 |  |  | 535.99 | 681.13 |  |  |
| 1 compound | 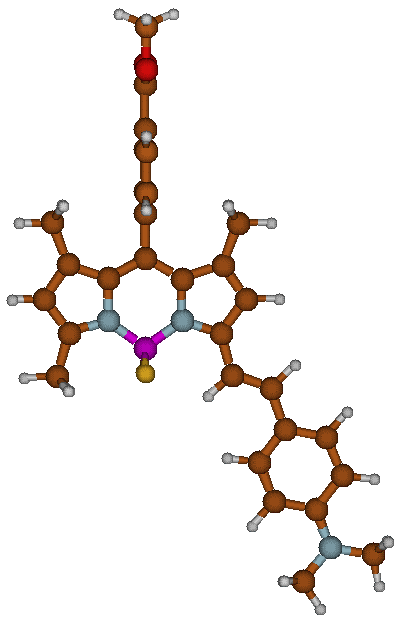 | 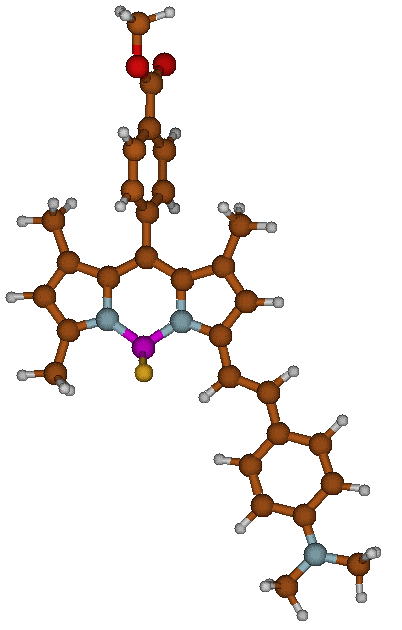 |  |  | 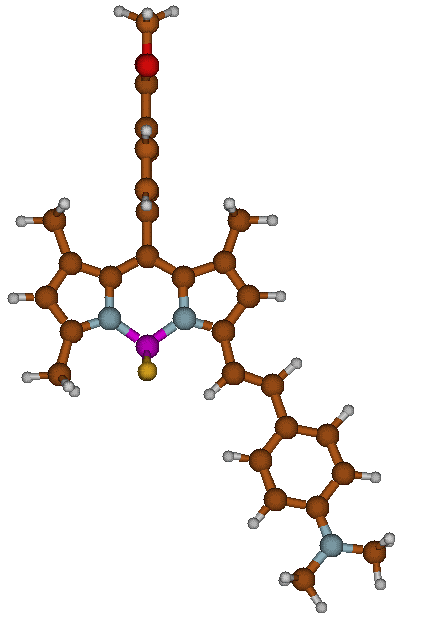 | 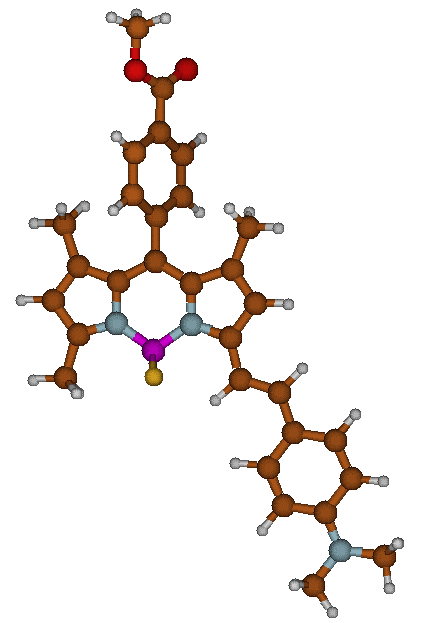 |  |  |
|  | 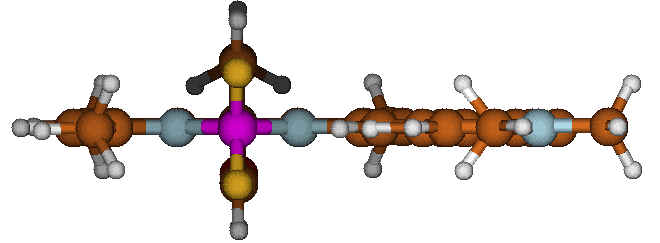 | 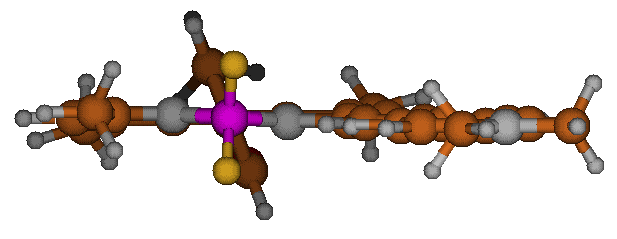 |  |  | 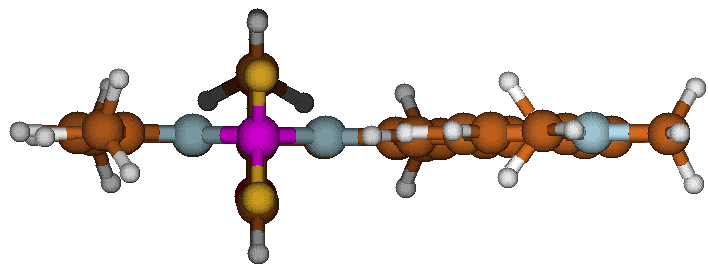 | 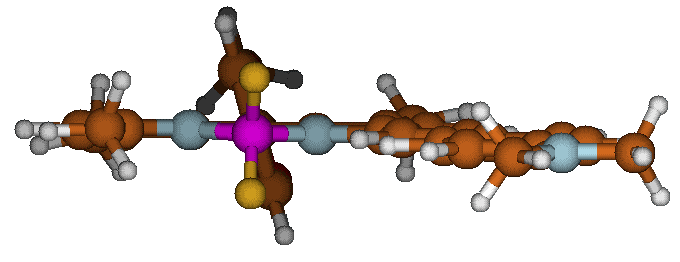 |  |  |
| E, hartree | -1296.22396 | -1296.22240 | -1296.21972 | -1296.21972 | -1297.15572 | -1297.15342 |  |  |
| λmax, nm | 428.02 | 478.84 | 501.35 | 501.29 | 434.99 | 497.47 |  |  |
| 2 compound | 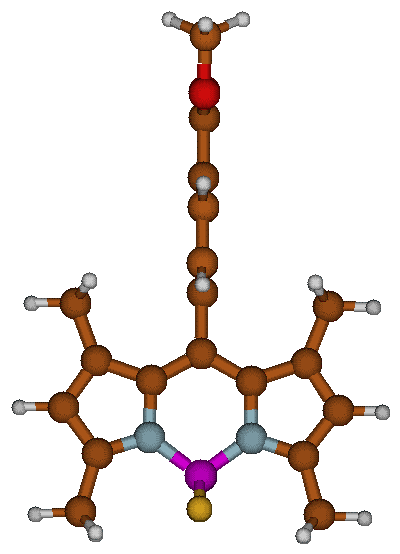 | 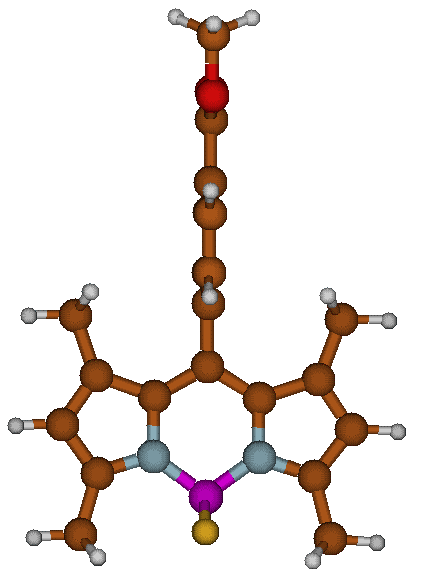 | 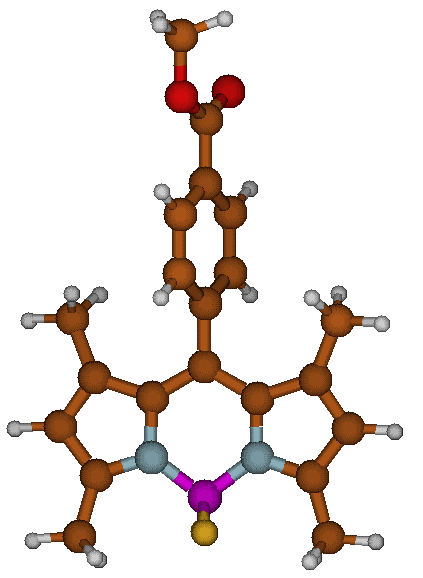 | 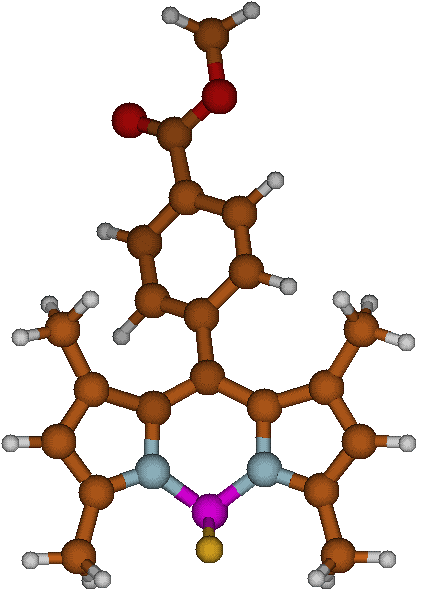 | 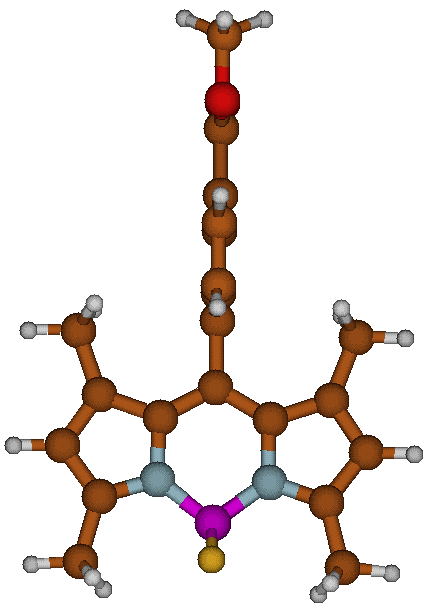 | 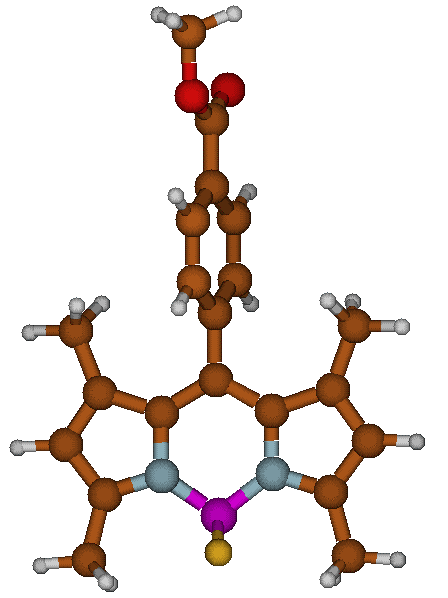 |  |  |
|  | 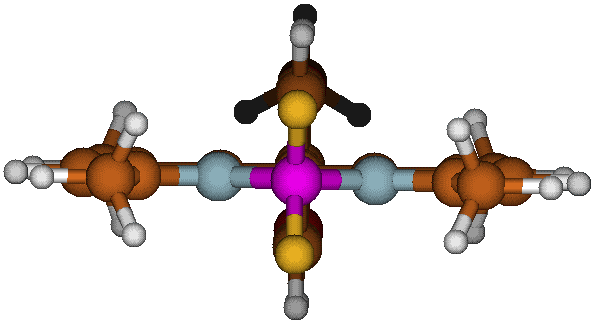 | 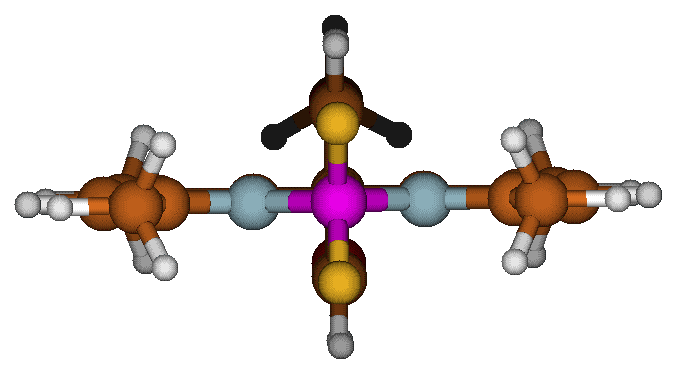 | 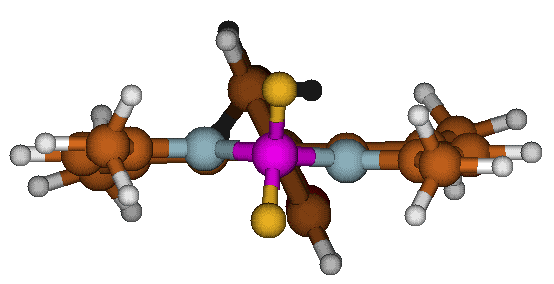 | 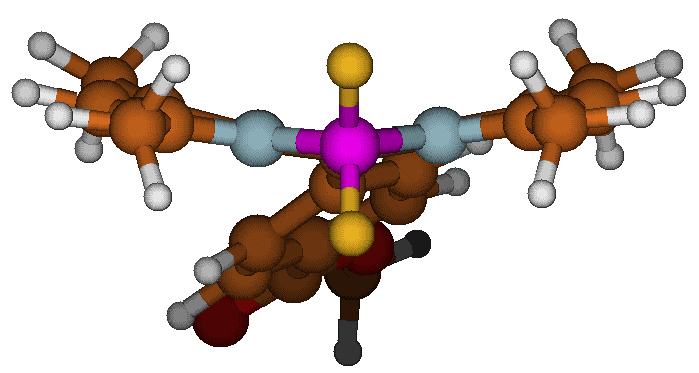 | 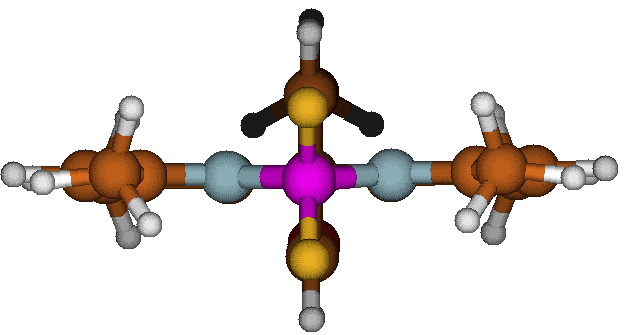 | 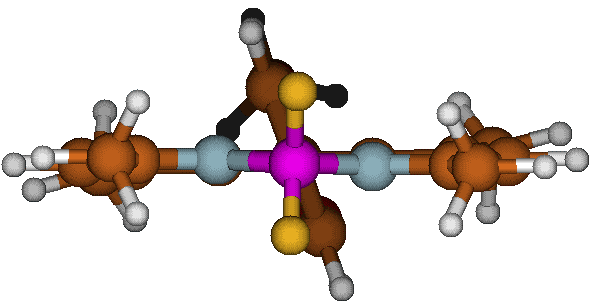 |  |  |
| E, Hartree | -1739.37355 | -1739.36960 |  |  | -1740.61753 | -1740.61348 | -1740.61348 | -1740.61354 |
| λmax, nm | 509.64 | 616.17 |  |  | 492.18 | 594.00 | 593.99 | 593.89 |
| 3 compound | 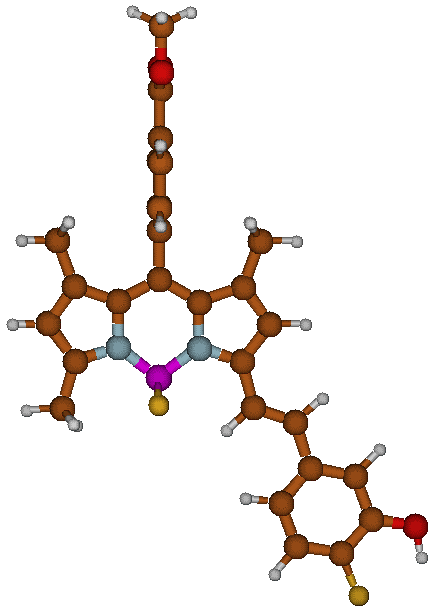 | 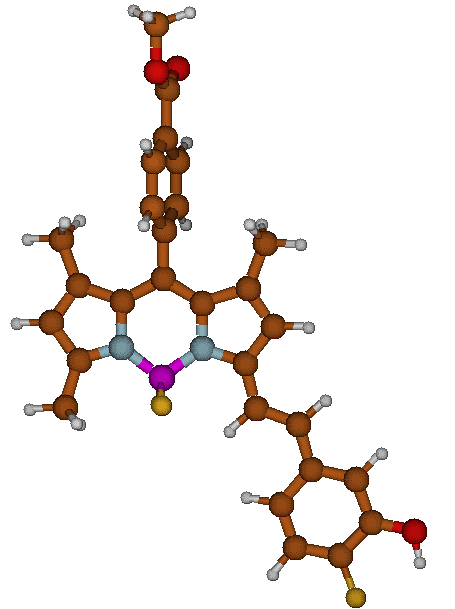 |  |  | 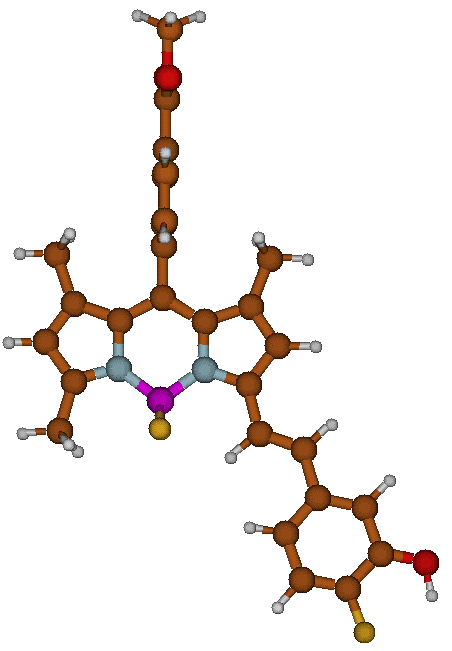 | 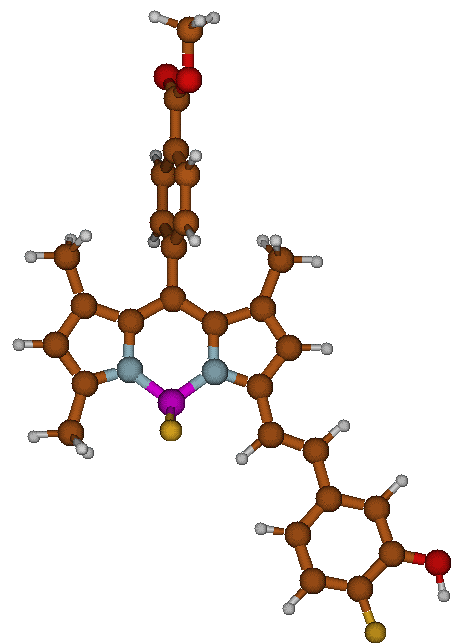 | 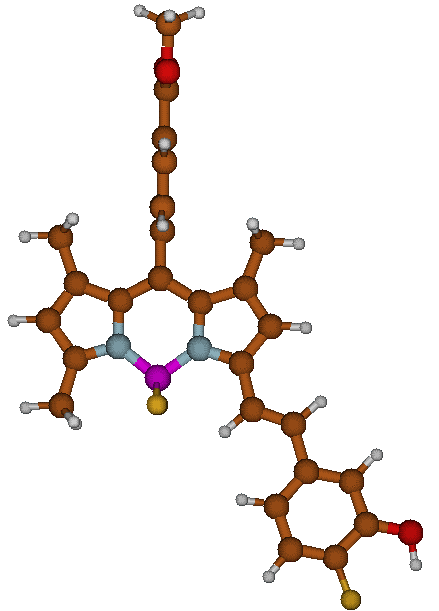 | 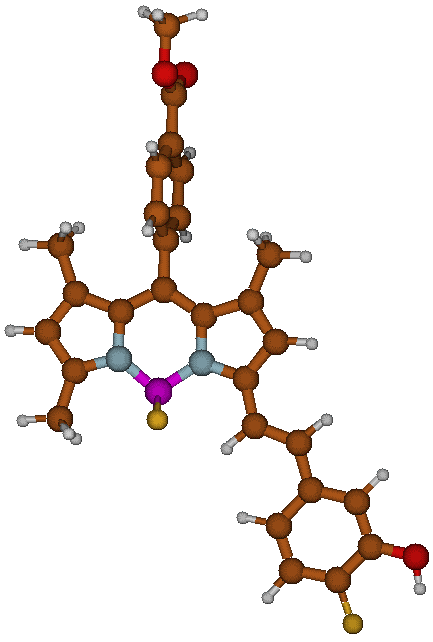 |
|  | 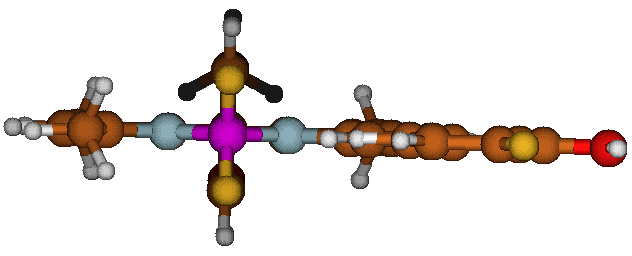 | 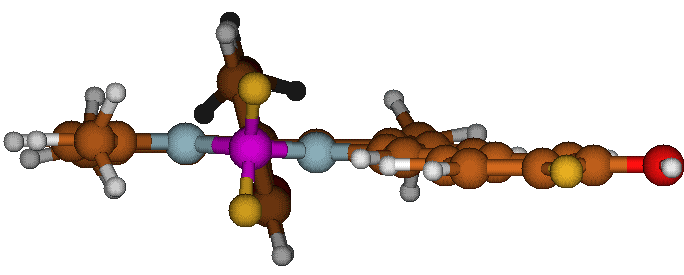 |  |  | 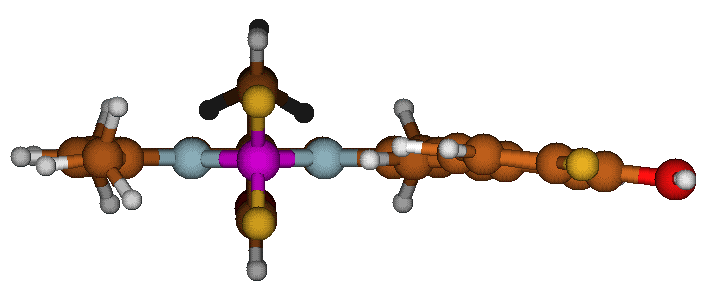 | 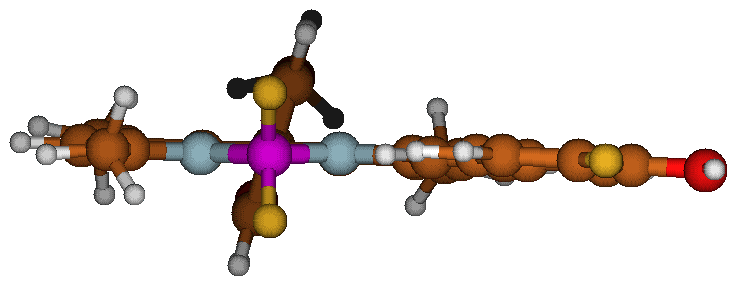 | 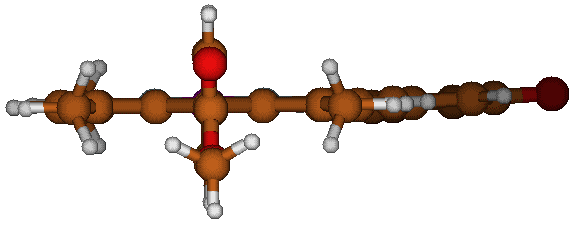 | 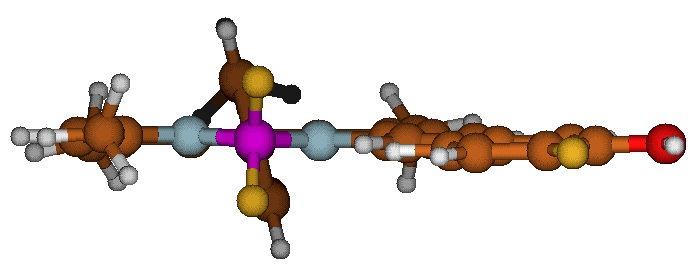 |

| Table S3. Calculated structures, spectral and energetic parameters of studied compounds by PBE0 and M062X methods in cyclohexane. | | | | | | | |
| --- | --- | --- | --- | --- | --- | --- | --- |
|  | PBE0 | | | M062X | | | |
|  | Ground state | Excited state | | Ground state | Excited state | | |
| E, Hartree | -1698.87943 | -1698.87436 | -1698.87436 | -1700.10864 | -1700.10404 | -1700.10415 |  |
| λmax, nm | 570.61 | 641.55 | 641.53 | 526.82 | 587.53 | 586.26 |  |
| 1 compound | 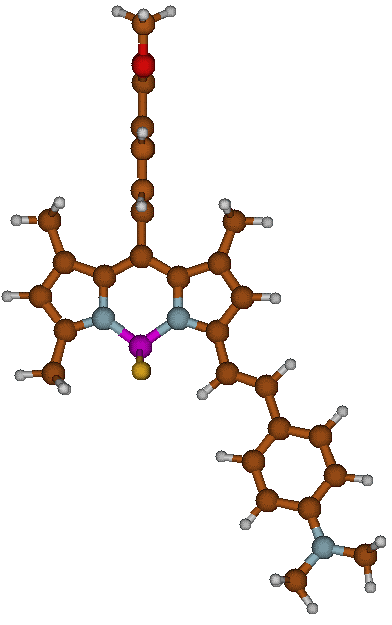 | 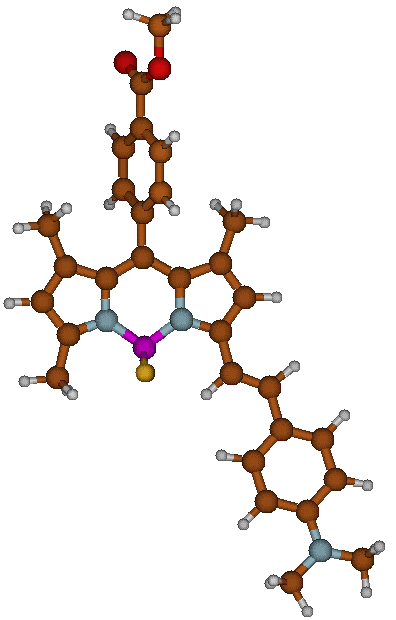 | 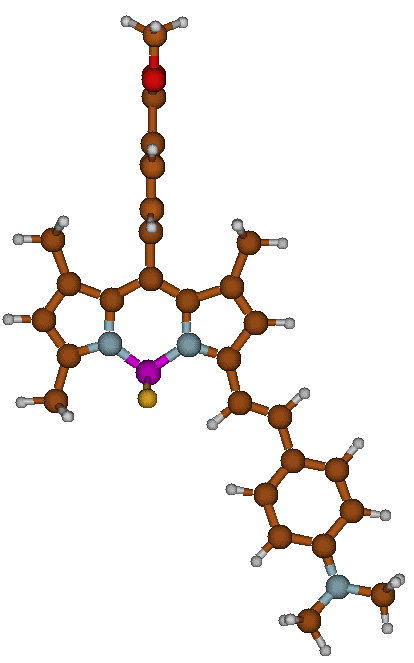 | 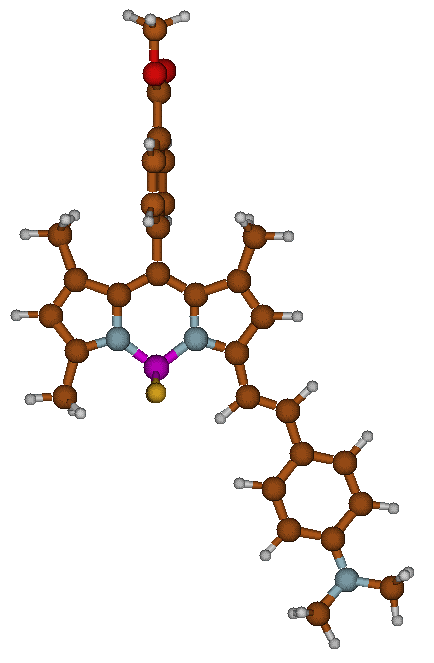 | 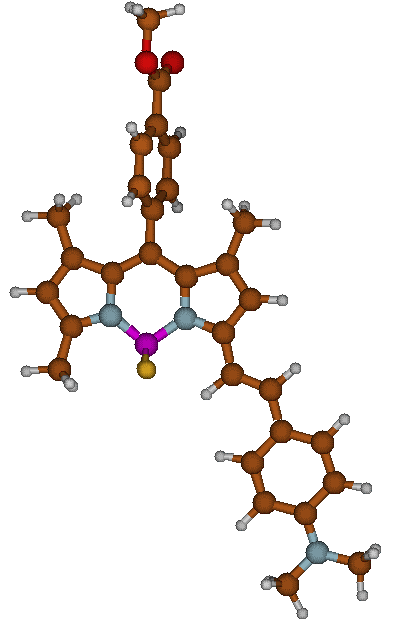 | 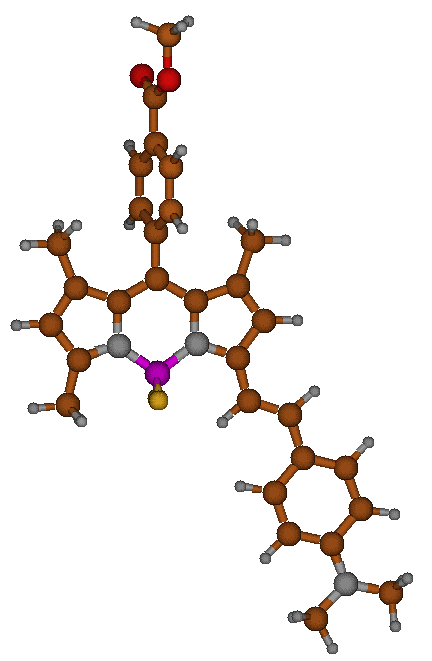 |  |
|  | 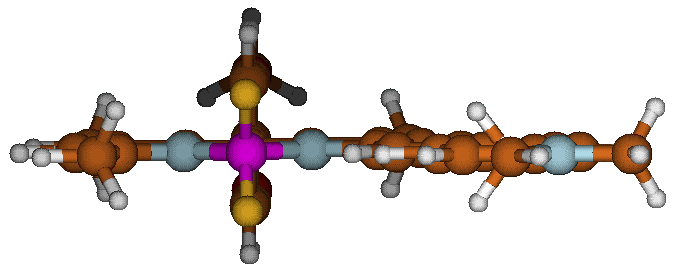 | 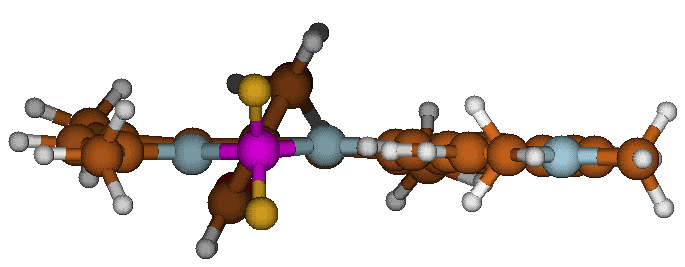 | 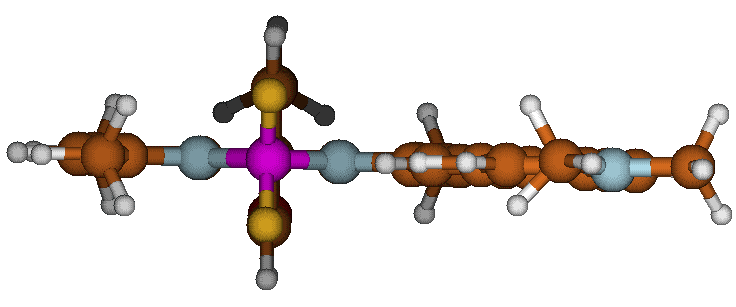 | 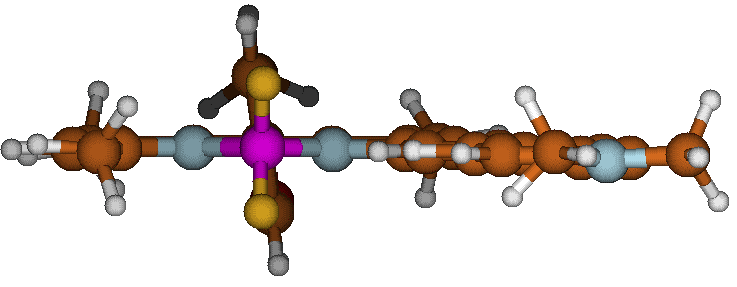 | 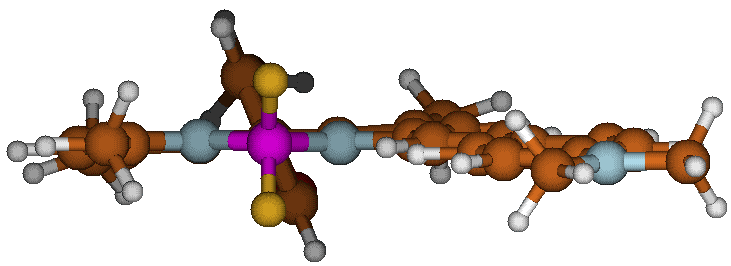 | 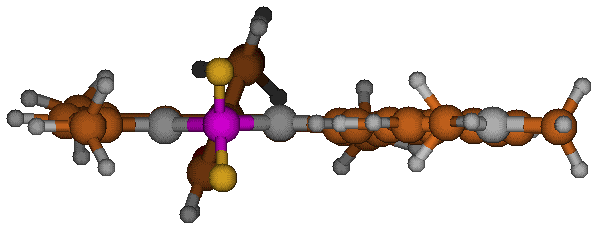 |  |
| E, Hartree | -1296.21474 | -1296.20821 | -1296.20821 | -1297.14657 | -1297.14370 | -1297.14369 |  |
| λmax, nm | 429.15 | 481.67 | 481.65 | 436.88 | 461.47 | 461.50 |  |
| 2 compound | 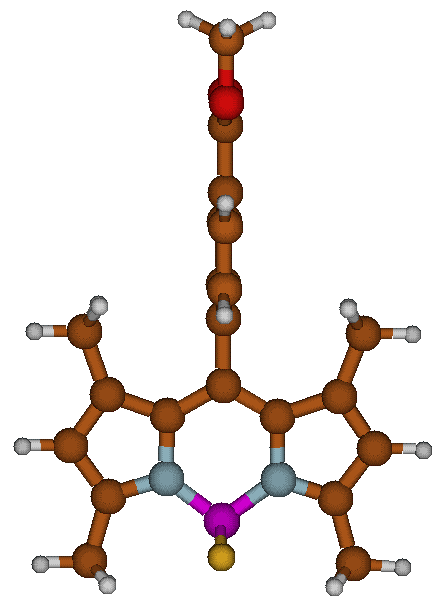 | 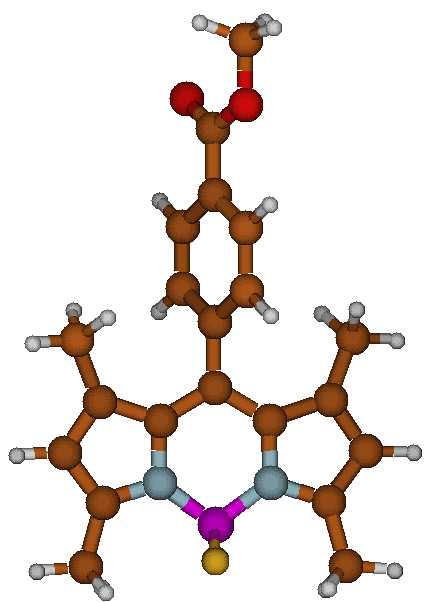 | 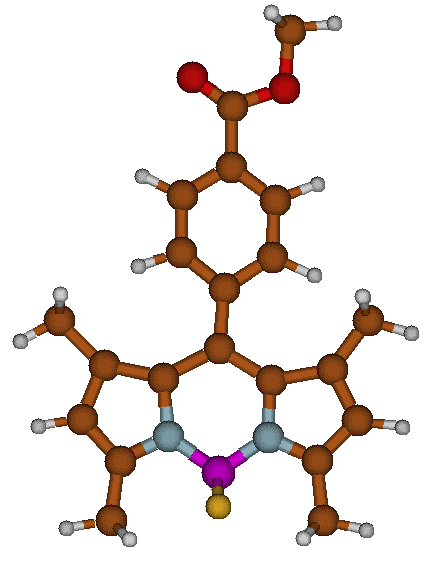 | 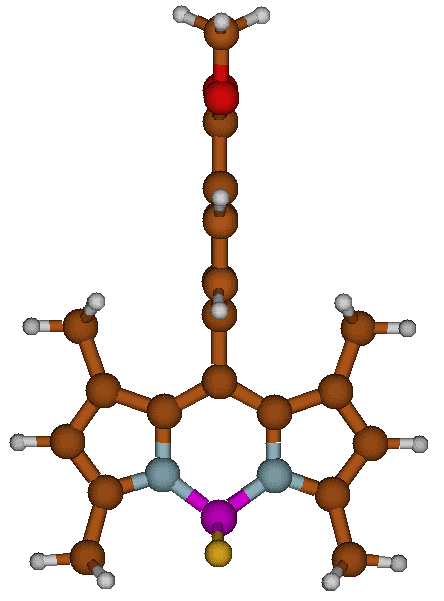 | 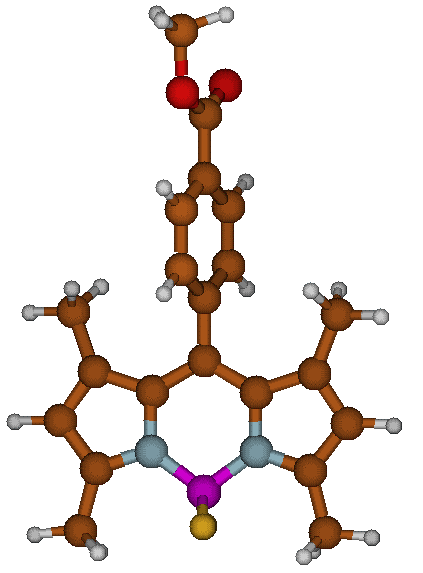 | 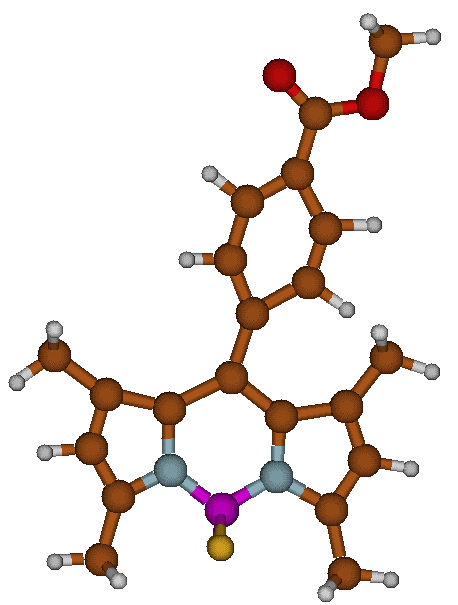 |  |
|  | 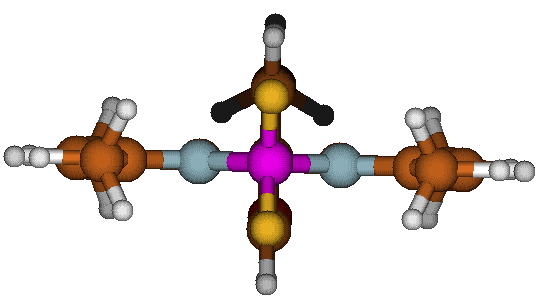 | 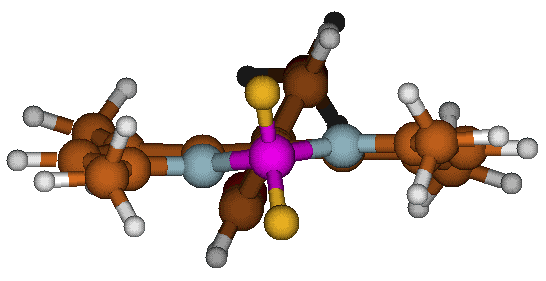 | 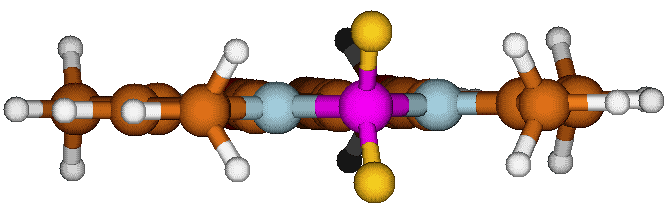 | 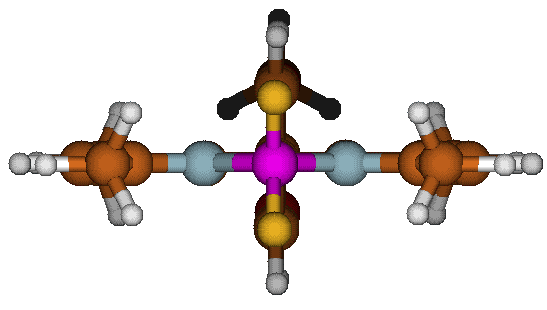 | 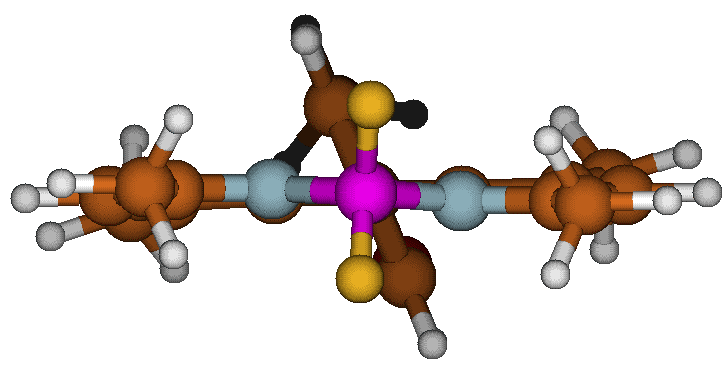 | 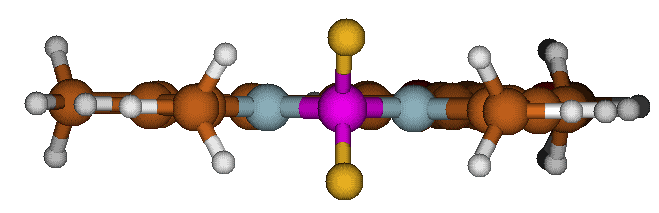 |  |
| E, Hartree | -1739.36115 | -1739.35608 | -1739.35609 | -1740.60523 | -1740.60163 | -1740.60163 | -1740.60166 |
| λmax, nm | 510.35 | 565.82 | 565.50 | 493.17 | 532.63 | 532.63 | 532.62 |
| 3 compound | 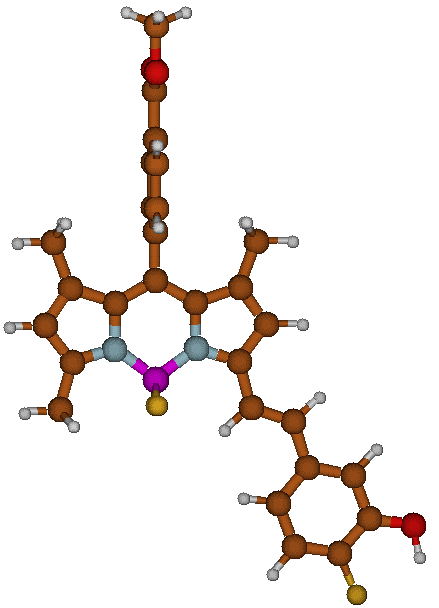 | 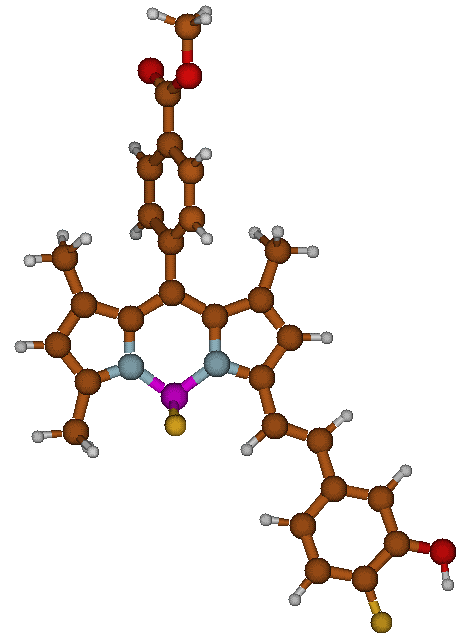 | 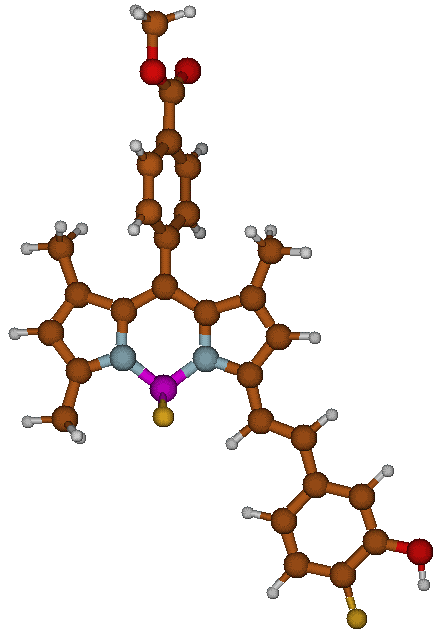 | 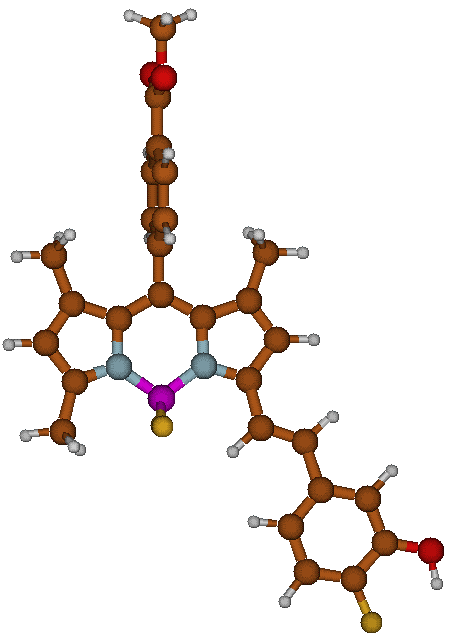 | 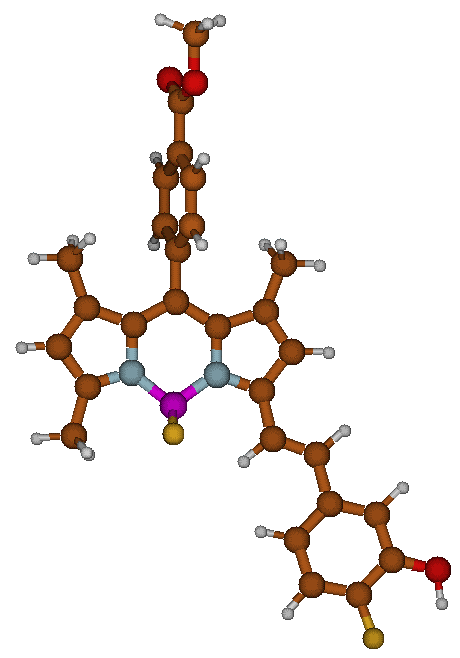 | 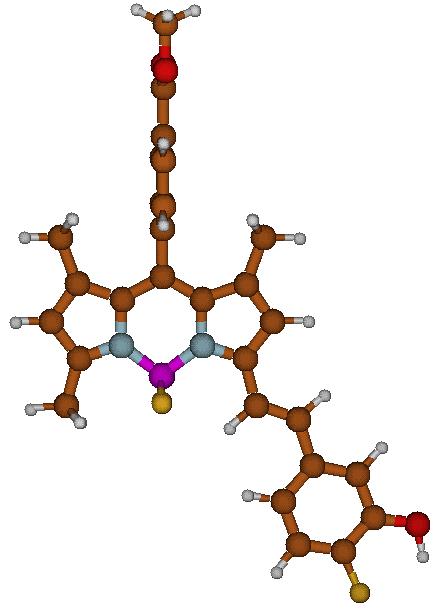 | 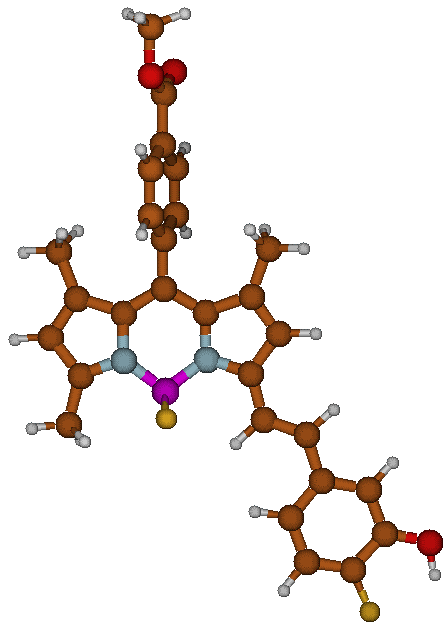 |
|  | 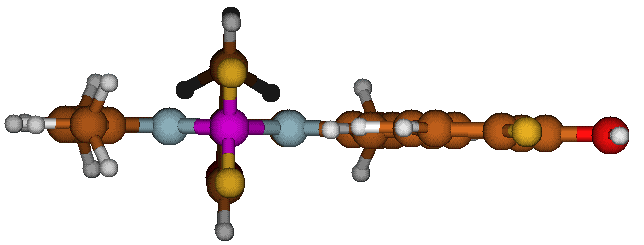 | 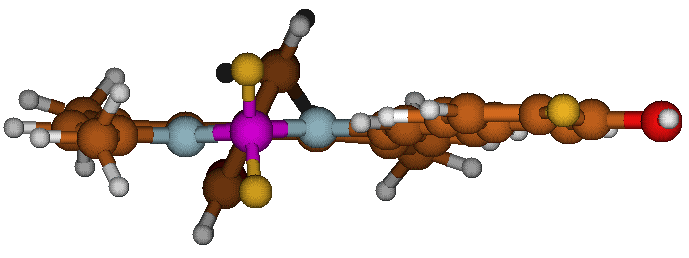 | 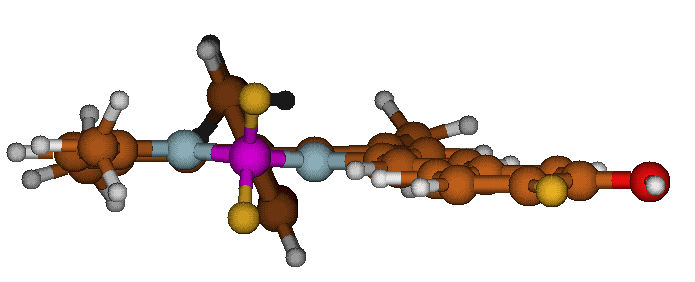 | 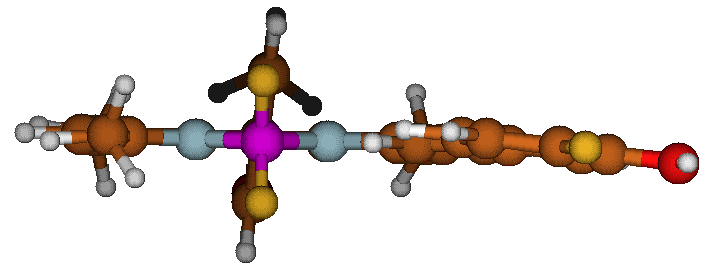 | 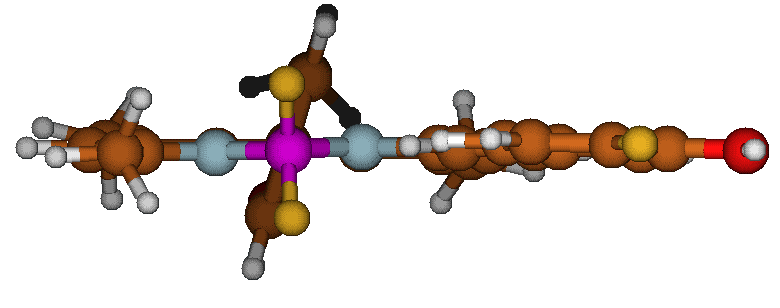 | 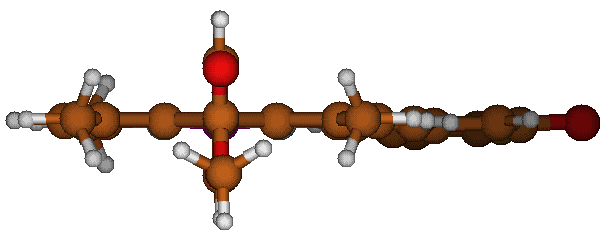 | 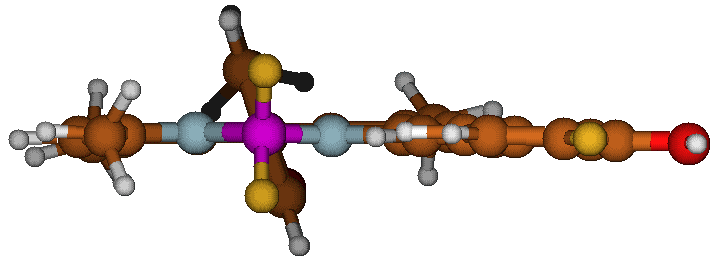 |

| Table S4. Calculated structures, spectral and energetic parameters of studied compounds by PBE0 and M062X methods in 1-butanol. | | | | | | | | |
| --- | --- | --- | --- | --- | --- | --- | --- | --- |
|  | PBE0 | | | | M062X | | | |
|  | Ground state | Excited state | | | Ground state | Excited state | | |
| E, Hartree | -1698.89171 | -1698.88796 |  |  | -1700.12050 | -1700.11538 |  |  |
| λmax, nm | 579.99 | 702.25 |  |  | 534.18 | 669.38 |  |  |
| 1 compound | 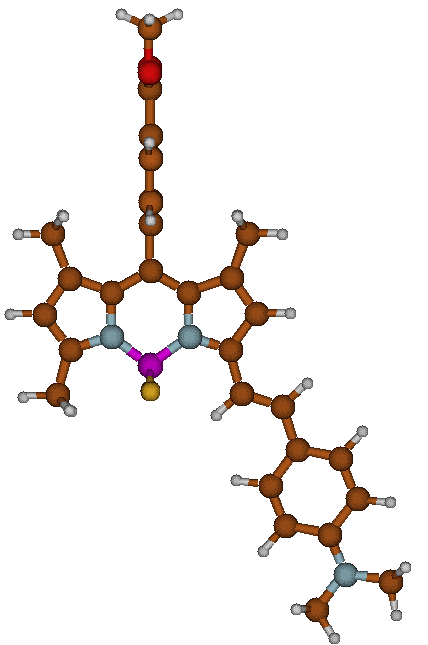 | 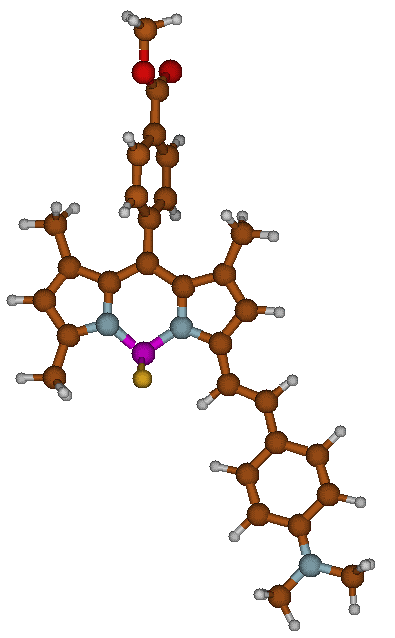 |  |  |  |  |  |  |
|  |  |  |  |  |  |  |  |  |
| E, Hartree | -1296.22302 | -1296.22148 | -1296.21856 | -1296.21856 | -1297.15478 | -1297.15245 | -1297.15245 |  |
| λmax, nm | 427.47 | 474.56 | 498.69 | 498.67 | 434.41 | 493.30 | 493.29 |  |
| 2 compound |  |  |  |  |  |  |  |  |
|  |  |  |  |  |  |  |  |  |
| E, Hartree | -1739.37229 | -1739.36827 | -1739.36834 | -1739.36827 | -1740.61631 | -1740.61229 | -1740.61229 | -1740.61233 |
| λmax, nm | 508.75 | 609.75 | 609.48 | 609.75 | 491.38 | 587.01 | 587.01 | 586.96 |
| 3 compound |  |  |  |  |  |  |  |  |
|  |  |  |  |  |  |  |  |  |

Figure S1. Dependencies of calculated λ_abs_ and λ_em_ parameters by PBE0 and M062X method with LR (solid line) and SS (dashed line) approaches versus λ_abs_ and λ_em_ experimental data of studied compounds.

|  | PBE0/6-31+G(d,p) | M062X/6-31+G(d,p) |
| --- | --- | --- |
| 1 compound |  |  |
| 2 compound |  |  |
| 3 compound |  |  |

Figure S2. Representation of ground/excited state and transition energies (a. u.) obtained by LR (solid line) and SS (dashed line) approaches of studied compounds in toluene.
